# Impaired Muscular Fat Metabolism in Juvenile Idiopathic Arthritis in Inactive Disease

**DOI:** 10.3389/fphys.2019.00528

**Published:** 2019-05-01

**Authors:** Emmanuelle Rochette, Pierre Bourdier, Bruno Pereira, Stéphane Echaubard, Corinne Borderon, Nicolas Caron, Aurélie Chausset, Daniel Courteix, Solenne Fel, Justyna Kanold, Justine Paysal, Sébastien Ratel, Nadège Rouel, Catherine Sarret, Daniel Terral, Alexandra Usclade, Etienne Merlin, Pascale Duché

**Affiliations:** ^1^Centre Hospitalier Universitaire de Clermont-Ferrand, Clermont-Ferrand, France; ^2^CIC 1405, Unité CRECHE, INSERM, Université Clermont Auvergne, Clermont-Ferrand, France; ^3^Laboratoire des Adaptations Métaboliques en Conditions Physiologiques et Physiopathologiques, Université Clermont Auvergne, Clermont-Ferrand, France; ^4^Centre de Recherche en Nutrition Humaine d’Auvergne, Clermont-Ferrand, France; ^5^Délégation à la Recherche Clinique et à l‘Innovation, Centre Hospitalier Universitaire de Clermont-Ferrand, Clermont-Ferrand, France; ^6^INRA, UMR 1019 UNH, ECREIN, Université Clermont Auvergne, Clermont-Ferrand, France; ^7^Laboratoire Impact de l’Activité Physique sur la Santé, Université de Toulon, Toulon, France

**Keywords:** pediatric, physical activity, inflammation, fat oxidation, metabolism

## Abstract

**Objectives:** The objective of this study was to evaluate muscular metabolic function in children with inactive juvenile idiopathic arthritis (JIA).

**Methods:** Fifteen children with inactive JIA and fifteen healthy controls were matched by sex, biological age, and Tanner stage. Participants completed a submaximal incremental exercise test to determine their fat and carbohydrate oxidation rates.

**Results:** Between the two groups, heart rate values and carbohydrate oxidation rates were the same, regardless of the relative intensity of exercise. Lipid oxidation rates were lower in JIA patients, regardless of the percentage of VO_2_ peak (*p* < 0.05). Respiratory exchange ratios beyond 50% of VO_2_ peak were higher in patients with JIA (*p* < 0.05). Respective maximal fat oxidation rates (MFO) for controls and children with JIA were 218.7 ± 92.2 vs. 157.5 ± 65.9 mg ⋅ min^-1^ (*p* = 0.03) and 4.9 ± 1.9 vs. 3.4 ± 1.2 mg ⋅ min^-1^ ⋅ kg^-1^ (*p* = 0.04). There was no difference between the two groups in heart rate, percentage of VO_2_ peak, or power of exercise to achieve MFO. Controls reached their MFO at an exercise power significantly higher than did JIA subjects (42.8 ± 16.8 and 31.9 ± 9.8 W, *p* = 0.004).

**Conclusion:** Children with JIA show metabolic disturbance during exercise, even when the disease is considered inactive. This disturbance is seen in a lower lipid oxidation rate during submaximal exercise.

## Introduction

In juvenile idiopathic arthritis (JIA), the most common rheumatoid disorder in pediatrics, an elevation of proinflammatory cytokines, such as IL-1β, IL-6, IL-8, or TNF-α, has been demonstrated in both the serum and synovial fluid of patients with JIA (Gorczyca et al., 2017). The highest cytokine levels were usually observed in the active phase of the disease, but they also stayed high in the clinical remission state (de Jager et al., 2007; Macaubas et al., 2009). However, the levels of these inflammatory markers are not used as a criterion for inactivity, which is mostly inferred from a defined set of clinical observations (Wallace et al., 2011).

Despite progress in anti-inflammatory treatments designed to target certain pro-inflammatory cytokines, including TNF-α (etanercept, adalimumab, infliximab), only half of patients achieve full and permanent remission (Bohr et al., 2016). Children and adolescents with chronic rheumatoid diseases are thus less physically active because of pain, joint limitations induced by their disease, and increased fatigue (Bohr et al., 2015; Armbrust et al., 2016; Bos et al., 2016). This behavior is accentuated during the active phases of the disease, but also persist in patients who are controlled and in those with inactive disease. The physical abilities of these children are thus impaired, and the literature reports atrophy and muscle weakness. They also have reduced aerobic and anaerobic abilities compared to healthy children (Roth et al., 2004; Lelieveld et al., 2007; van Brussel et al., 2007; van Pelt et al., 2012; Hulsegge et al., 2014).

The anti-inflammatory treatments used to control JIA have observable adverse effects, such as dyslipidemia, a major risk factor for the development of atherosclerosis (Yeh et al., 2014; Kraakman et al., 2016). Proinflammatory cytokines that are involved in JIA, like TNF-α, also play a significant role in tissue insulin resistance, lipid metabolism, and muscle health (Wu and Ballantyne, 2017). However, impaired metabolic control of lipid and glucose homeostasis predisposes an individual to cardiometabolic diseases (Fernández-Hernando et al., 2013). Children with JIA therefore form a population at increased risk of developing cardiovascular disease in adulthood.

As evaluated by calorimetry, maximal fat oxidation rate during incremental exercise is tightly correlated with mitochondrial function and might be a marker of metabolic fitness (Brun et al., 2012). An ability to oxidize lipids during exercise likely reflects a profile of metabolic fitness, which is correlated with the physiological status of muscles (Brun et al., 2012). In a context of chronic inflammation and physical deconditioning, children and adolescents with JIA should present impaired oxidation of energy substrates during physical exercise compared to healthy children. This would result in reduced lipid oxidation and prompter carbohydrate oxidation compared to children with no inflammatory disorder.

To evaluate metabolic responses, we used an indirect calorimetry test to evaluate substrate oxidation during submaximal incremental exercise in healthy sex- and age-matched peers compared to children with JIA who had not been treated with anti-TNF-α and who were in clinical remission according to the criteria of Wallace et al.

## Materials and Methods

### Patients

Fifteen children aged 7–18 years with inactive JIA (according to the International League of Associations for Rheumatology criteria) and fifteen healthy controls were enrolled in the study. Inactive disease status was evaluated according to the American College of Rheumatology’s criteria (Wallace et al., 2011). Subjects in the two groups were matched for age, pubertal stage (according to the Tanner stage), and sex.

Subjects were excluded if they had a physician-diagnosed infection, had received oral corticosteroids within the previous 3 months, or had received anti-TNF-α blockade treatment in the past. All other treatments had been administered for at least 3 months at the time of evaluation. Physical activity level (PAL) was determined according to the International Physical Activity Questionnaire for Adolescents (IPAQ-A) (Ottevaere et al., 2011), and individuals were classified into one of three levels of physical activity (PA): low, moderate, or high.

This study was carried out in accordance with the recommendations of the Comité de Protection des Personnes (CPP) Sud-Est VI, with written informed consent given by all subjects in compliance with the Declaration of Helsinki. The protocol was approved by the Comité de Protection des Personnes (CPP) Sud-Est VI (clinical trial number NCT 02977416). All participants and their parents gave their informed consent and were free to withdraw from the study at any time. All the subjects were followed between January 2017 and March 2018 at the pediatric unit of the Clermont-Ferrand University Hospital, France.

### Experimental Procedure

Participants were asked to refrain from consuming any food or liquid other than water in the 3 h before the visit. They also avoided calorie-rich food and refrained from strenuous physical activity for at least 24 h beforehand. After sitting quietly for 20 min, the subjects performed, to the point of volitional fatigue, a graded exercise test on an electromagnetically braked cycle ergometer with continuous gas collection and heart rate monitoring. Following a 2-min warm-up consisting of unloaded pedaling, subjects at Tanner stages 1 and 2 started cycling at 10 W, and their work rate was increased by 10 W every 3 min. Subjects at Tanner stages 3 and 4 started at 20 W, and their work rate was increased by 15 W every 3 min. When heart rate was unstable, this stage was extended for up to 5 min to obtain a stable heart rate to within ± 5 beats. When the respiratory exchange ratio (RER) was greater than or equal to 1.00 – indicating the absence of fat oxidation – work rate was increased by the same increments at 1-min intervals until volitional fatigue was reached. The VO_2_ peak was considered to have been reached when the RER was greater than or equal to 1.05 and the subject achieved his or her age-predicted maximal heart rate (HR_max_: 220 – age), according to the methodology validated by Riddell et al. (2008).

### Measurements

All the tests were performed on a Cyclus 2 ergometer (RBM Elektronik-Automation GmbH, Leipzig, Germany). O_2_ consumption (VO_2_) and CO_2_ production (VCO_2_) were measured breath by breath through a mask connected to an O_2_ and CO_2_ analyzer (MetaMax 3b, Cortex Biophysik, Leipzig, Germany).

Ventilatory parameters were averaged every minute during the submaximal exercise test and the subsequent 10-min recovery period. Heart rate was monitored continuously throughout the duration of the tests (Polar RS800cx monitor, Polar, Finland).

### Data Analysis

Indirect calorimetry is the recognized standard method to quantify substrate oxidation rates at rest and during exercise (Frayn, 1983). The VO_2_ and VCO_2_ values were averaged over the last minute of each work rate, the results then being used to calculate fat oxidation over a wide range of exercise intensities for each subject (Achten et al., 2002) using Péronnet and Massicotte’s equation (Péronnet and Massicotte, 1991): lipids (mg ⋅ min^-1^) = 1.6946 × VO_2_ - 1.7012 × VCO_2_; carbohydrate (CHO) (mg ⋅ min^-1^) = 4.585 VCO_2_ - 3.2255 VO_2_.

For each individual, a best-fit polynomial curve was constructed for fat and CHO oxidation rate (expressed as mg ⋅ min^-1^) vs. exercise intensity (expressed as a percentage of the VO_2_ peak). Each individual curve was then used to determine the peak fat oxidation rate and the exercise intensity associated with the maximal fat oxidation (MFO) rate (Achten et al., 2002).

### Statistical Considerations

Sample size was estimated according to (i) the CONSORT 2010 statement, extension to randomized pilot and feasibility trials (Eldridge et al., 2016) and (ii) Cohen’s recommendations (Statistical Power Analysis for the Behavioral Sciences, 2nd ed., NJ, United States, Lawrence Erlbaum, 1988), which define effect-size bounds as follows: small (ES: 0.2), medium (ES: 0.5) and large (ES: 0.8, “grossly perceptible and therefore large”). With 15 patients per group, we have an effect-size of around 1 for a two-sided type I error at 5% and a statistical power at 80%, with respect to the primary endpoint, namely maximal lipid oxidation (MFO) rate. Statistical analyses were performed using Stata software version 13 (StataCorp., College Station, TX, United States). Tests were two-sided with the type-I error set at 5%. The continuous data were expressed as mean ± standard deviation (SD) or as median (interquartile range) according to the statistical distribution. Assumption of normality was assessed with the Shapiro–Wilk test. Comparisons between groups (children with JIA vs. healthy controls) concerning the non-repeated quantitative parameters (age, body mass index, disease duration, VO_2_ peak, VO_2_ peak ⋅ kg^-1^ of body weight, MFO) were performed using the Student *t*-test or the Mann–Whitney test when assumptions required for the *t*-test were not met. Homoscedasticity was analyzed using the Fisher-Snedecor test. To account for the between- and within-patient variability due to several measures being taken for the same subject, random-effects models for the correlated data were then run rather than the usual statistical tests, as these would have been inappropriate due to an unverified assumption of independence. Time-point evaluations, the group of subjects (children with JIA vs. healthy controls), and their interactions were considered as fixed effects. The subject was considered a random effect (slope and intercept). A Sidak *post hoc* test was applied to correct the type-I error due to multiple comparisons. The normality of the residuals from these models was studied as described above using the Shapiro-Wilk test. When appropriate, the data were log-transformed to achieve normality of the dependent endpoint.

## Results

The subjects’ characteristics are summarized in Table 1. There was no significant difference between our patients with JIA and our controls in terms of BMI, VO_2_ peak, VO_2_ peak per kilogram of body weight, rest metabolism, or physical activity level. In patients with JIA, disease duration was 72.8 ± 48.6 months. Of the 15 patients, only one was treated with NSAIDs, six with methotrexate.

**Table 1:** Participants’ characteristics.

		Healthy
	JIA	controls	*p*
*n*	15	15	
Sex (*n*; male/female)	3/12	3/12	
Age (years) mean ± SD	13.7 ± 3.3	13.4 ± 3.5	0.85
Tanner stage (*n*; I–II/III–IV)	5/10	5/10	
Body mass (kg) mean ± SD	47.4 ± 13.3	47.0 ± 14.4	0.94
Height (cm) mean ± SD	155.7 ± 15.7	153.3 ± 16.4	0.70
BMI (kg/m^2^) mean ± SD	19.1 ± 2.7	19.4 ± 3.1	0.74
JIA subtype (*n*)		–	
oJIA	8	–	
pJIA RF-	3	–	
ERA	2	–	
psoriatic	1	–	
Undifferentiated	1	–	
Disease duration (months) mean ± SD	72.8 ± 48.6	–	
DMARDs (*n*)		–	
NSAIDs	1		
MTX	6		
IPAQ score (*n*)			
Low level of physical activity	3	3	0.17
Moderate level of physical activity	7	6	
High level of physical activity	5	6	
VO_2_ peak (ml/min) mean ± SD	1486.0 ± 523.2	1695.5 ± 622.3	0.45
VO_2_ peak/body mass (ml/kg/min) mean ± SD	32.1 ± 7.9	36.7 ± 8.5	0.13
Rest metabolism (kcal/day) mean ± SD	1560.3 ± 367.2	1885.5 ± 566.7	0.11

*JIA, juvenile idiopathic arthritis; oJIA, oligoarticular JIA; pJIA RF-, rheumatoid factor-negative (RF-) polyarticular JIA; ERA, enthesitis-related arthritis; psoriatic, psoriatic JIA; DMARDs, disease-modifying antirheumatic drugs; MTX, methotrexate; NSAIDs, non-steroidal anti-inflammatory drugs; IPAQ, International Physical Activity Questionnaire.*

**FIGURE 1:**
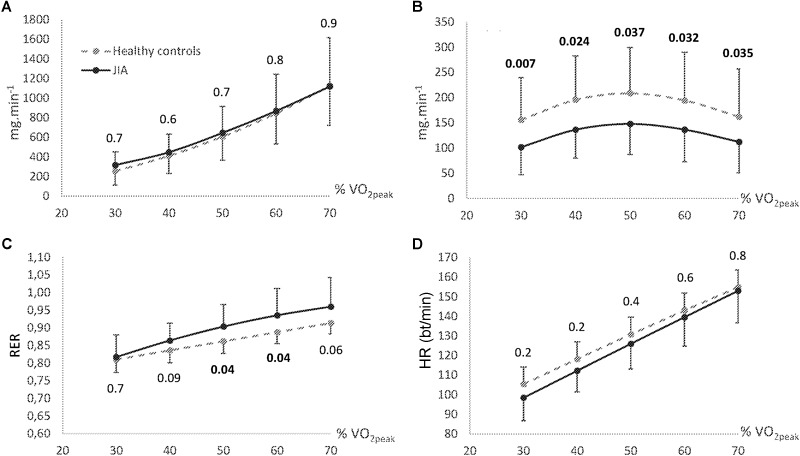
Carbohydrates (CHO) **(A)** and fat **(B)** oxidation rate, respiratory exchange ratio (RER) **(C)**, and heart rate **(D)** according to percentage of VO_2_ peak. Data are means ± 95% CI. Bt, beats; JIA, juvenile idiopathic arthritis.

The oxidation rates of lipids and carbohydrates as a function of the percentage of VO_2_ peak are shown in Figure 1. For exercise intensities corresponding to the same percentages of VO_2_ peak, the carbohydrate oxidation rate was the same in the two groups (Figure 1A). However, lipid oxidation rates were statistically lower from the exercise intensities corresponding to 30% of VO_2_ peak up to those corresponding to 70% of VO_2_ peak (Figure 1B). Regarding the respiratory exchange ratio (RER), the intensities corresponding to 30 and 40% VO_2_ peak showed no difference between the two groups (*p* = 0.7 and 0.09) (Figure 1C). By contrast, beyond 50% of VO_2_ peak, there was a statistically significant increase in the value of the RER in patients with JIA. Finally, the heart rate values were the same for the two groups, regardless of the intensity of exercise (Figure 1D).

**FIGURE 2:**
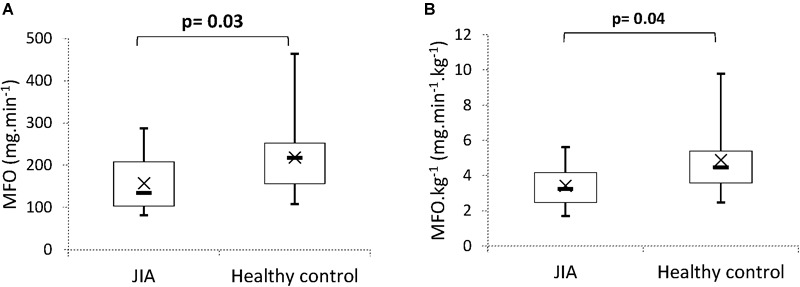
Comparison of maximal fat oxidation (MFO) **(A)** and maximal fat oxidation per kg of total body mass **(B)**. Boxes represent interquartile ranges and whiskers give minimum and maximum values. Data are means (×) and medians (–). JIA, juvenile idiopathic arthritis.

The maximal lipid oxidation rate (MFO) was significantly different between the two groups. For controls and children with JIA, the respective MFO was: 218.7 ± 92.2 vs. 157.5 ± 65.9 mg ⋅ min^-1^ (*p* = 0.03) and 4.9 ± 1.9 vs. 3.4 ± 1.2 mg ⋅ min^-1^ ⋅ kg^-1^ (*p* = 0.04) (Figure 2).

**FIGURE 3:**
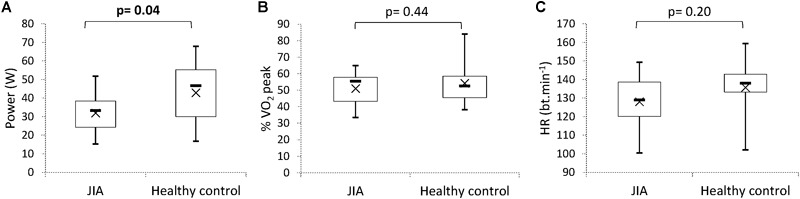
Comparison of power **(A)**, % VO_2_ peak **(B)**, and heart rate (HR) **(C)** at the maximal fat oxidation rate (MFO). Boxes represent interquartile ranges and whiskers give minimum and maximum values. Data are means (×) and medians (–). Bt, beats; JIA, juvenile idiopathic arthritis.

Heart rate, percentage of VO_2_ peak, and power of exercise to achieve MFO are shown in Figure 3. Between controls and patients with JIA, there was no difference in heart rate (respectively, 135.7 ± 15.2 and 128.1 ± 13.4 beats ⋅ min^-1^, *p* = 0.2, Cohen’s *d* = 0.5) or percentage of VO_2_ peak (respectively, 54.3 ± 12.2 and 51.1 ± 9.4%, *p* = 0.4, Cohen’s *d* = 0.2) to reach MFO. However, controls achieved a significantly higher MFO at exercise power than subjects with JIA (42.8 ± 16.8 and 31.9 ± 9.8 W, respectively, *p* = 0.004).

## Discussion

Even when the disease was considered inactive, children with JIA showed a metabolic disturbance during submaximal exercise, with lower lipid oxidation rates than controls, regardless of exercise intensity, while there was no difference in carbohydrate oxidation rates. These observations imply a lower contribution from lipids to meeting energy demand, reflected by high RER values at low relative exercise intensities, which will result in greater fatigability in these children. The lipid oxidation system seems limited and insufficient to provide the required energy, and the carbohydrate proportion then becomes predominant at relatively low exercise intensities (40–50% VO_2_ peak). Consequently, we can expect patients with JIA to be more easily fatigable than healthy children and be unable to maintain high-intensity exercise. One of the limiting factors in lipid oxidation is the rapid turnover of enzymes involved in β-oxidation (Jeppesen and Kiens, 2012). The impaired energy metabolism in these children may be the consequence of mitochondrial dysfunction. Although this phenomenon has been studied only in systemic JIA, two teams have demonstrated a modification of mitochondrial gene expression that suggests that systemic JIA is not only an immunological disease, but also a metabolic disease involving mitochondrial disorders (Ishikawa et al., 2009; Omoyinmi et al., 2016). Whether this mitochondrial involvement is specific to systemic JIA or a non-specific systemic inflammation needs to be addressed. If it is a non-reversible physiological state involving dysregulation of signaling pathways or molecular functional changes (i.e., transporters, enzymes, etc.), this would imply that the metabolic impairment is constitutional in JIA. The question arises of whether the impaired energy metabolism is consecutive to chronic inflammation and deconditioning or part of the pathophysiology of this autoimmune disease. Since we matched the physical activity levels of our patients with JIA to those of controls, this impairment is likely due to the disease. A longitudinal follow-up of the oxidative profile of substrates from the diagnosis and comparison with other inflammatory diseases could help answer this question.

Half of our patients were still on medication (one being treated by non-steroidal anti-inflammatory drugs [NSAIDs] and six by methotrexate), and these treatments have a potential impact on energy metabolism. However, our results conflict with *in vitro* data that showed that methotrexate, by activation of AMPK, enhances lipid oxidation and glucose uptake in skeletal muscle (Pirkmajer et al., 2015). Nevertheless, in the profiles of the patients who were not under treatment, lipid oxidation rates remained lower than those of their paired controls [for controls and children with JIA not under treatment, the respective MFO ⋅ kg^-1^ was 5.5 ± 2.0 vs. 3.5 ± 1.1 mg ⋅ min^-1^ (*p* = 0.02), Supplementary Table 1]. Furthermore, compared to the controls, children with JIA reached lower exercise powers for the same exercise intensities relative to VO_2_ peak. This may be a reflection of lower muscle energy efficiency, lack of motor unit recruitment or autonomic nervous system dysfunction (Kuis et al., 1996). These results may also be related to insulin resistance induced by low-grade chronic inflammation and in particular higher levels of TNF-α. Skeletal muscle is an insulin-sensitive organ that plays a crucial role in maintaining systemic glucose homeostasis (Carnagarin et al., 2015). Inflammation and insulin resistance are closely related, and inflammatory cytokines such as TNF-α, IL-6, IL-1, and IL-8 may inhibit insulin signaling via multiple mechanisms (Wellen and Hotamisligil, 2005). However, in young JIA patients, there is no documentation of glucose intolerance or an increased HOMA insulin resistance index (Homeostasis Model Assessment of insulin resistance), a strong predictor for reduced glucose tolerance (Sinha et al., 2002). Nonetheless, when JIA is associated with obesity, it has been reported that the HOMA index increases (da Silva et al., 2010; Głowińska-Olszewska et al., 2013). Despite a high rate of inflammation, it can be assumed that adipocyte lipolysis is not different between our two groups and that it allows maintenance of muscular insulin sensitivity, and so does not adversely affect carbohydrate metabolism (Girousse et al., 2013). In line with our results, there is no difference in the carbohydrate oxidation rate, so there seems to be no impairment of glucose metabolism in the subjects of our study.

Finally, it is possible that this metabolic involvement results from an infra-clinical inflammation, which would mean that the criteria for defining an inactive disease are not optimal. As defined by Wallace et al. (2011), the criteria for an inactive disease are: no joints with active arthritis; no fever, rash, serositis, splenomegaly, or generalized lymphadenopathy attributable to JIA; no active uveitis; C-reactive protein (CRP) or erythrocyte sedimentation rate (ESR) level within normal limits; duration of morning stiffness less than 15 min; and a physician’s global assessment of disease activity score of best possible on the scale used. Only one biological criterion is thus used to define the activity of the disease; other biological biomarkers (such as inflammatory cytokine levels), might thus be relevant in the absence of clinical signs. Another possibility is that these children have subclinical inflammation and that the criteria used for inactivity of the disease do not reflect any real remission state. The exploration of these children’s energy metabolism during exercise could therefore be an indicator of subclinical activity of the disease.

However, impaired lipid metabolism may be the whole body’s adaptation to a state of less activity and low energy expenditure resulting from the low level of physical activity of children with JIA (Bohr et al., 2015; Bos et al., 2016). Lipid oxidation capacity is related to physical condition, itself related to levels of physical activity. In this case, the system could be remobilized by a physical activity program. Chronic physical activity is associated with an increase in the proportion of oxidized lipids during exercise and an improvement in the mitochondrial enzymatic capacities involved in β-oxidation (Tarnopolsky et al., 2007). Ongoing physical activity is also associated with an increase in insulin sensitivity because physical activity improves glucose transport and increases the expression or activity of entities involved in insulin-signaling pathways, such as protein B kinase (Akt) or AMPK (Benatti et al., 2018). Accordingly, prescription of regular physical activity could help correct this energy metabolism disturbance.

The limits of this work arise from the small size of the study population and the heterogeneity of this disorder in terms of subtypes and treatments used. More accurate data on body composition (assessed by dual-energy X-ray absorptiometry), physical activity levels (evaluated by actimetry), and inflammation (especially TNF-α and IL-6 levels) would need to be collected. In addition, data on glycemic and lipidemic blood markers could have told us whether any of our patients had dyslipidemia or an impairment of metabolic flexibility.

## Conclusion

Our results show that even when the disease is considered inactive, muscular metabolism is disturbed, suggesting a functional impairment at the muscle level in children with JIA. This impairment could have a long-term impact on the health of these children, especially for their cardiovascular system.

## Author Contributions

ER wrote the manuscript. PD and ER drafted the study design. ER and PB conducted the data acquisition and analysis. SE and EM performed the clinical assessments and data acquisition. BP performed the statistical analysis. PD and EM supervised the project. All authors contributed to the manuscript revision and read and approved the submitted version.

## Conflict of Interest Statement

The authors declare that the research was conducted in the absence of any commercial or financial relationships that could be construed as a potential conflict of interest. The reviewer CH declared a past co-authorship with several of the authors PD, EM, SE, BP, and ER to the handling Editor.
