# Supplementary material for: Impaired Muscular Fat Metabolism in Juvenile Idiopathic Arthritis in Inactive Disease
Source: Front Physiol. 2019 May 1;10:528. doi: 10.3389/fphys.2019.00528 (PMC6506786; doi:10.3389/fphys.2019.00528)
Supplement: Supplementary file 1 [file Table_1.docx]

**Supplementary table 1:** under treatment patients’characteristics

| age (years) | Sex | Body mass (Kg) | Height (cm) | BMI (kg/m²) | Tanner stage | JIA subtype | Disease duration (months) | DMARDs | IPAQ-score | VO_2_peak (ml/min) | VO_2_peak  /body mass (ml/kg/min) | Rest metabolism (kcal/day) | MFO (mg/min) | MFO/bm (mg/min/kg) | %VO_2_ at MFO | HR at MFO (Beat/min) | Power at MFO (W) |
| --- | --- | --- | --- | --- | --- | --- | --- | --- | --- | --- | --- | --- | --- | --- | --- | --- | --- |
| 17.5 | f | 48.6 | 160 | 19.0 | IV | oJIA | 100.4 | MTX | high | 1490.0 | 30.7 | 1285.9 | 209.8 | 4.3 | 57.4 | 149.2 | 38.5 |
| 16.0 | f | 61.0 | 174 | 20.1 | IV | pJIA RF− | 31.5 | MTX | low | 1420 | 23.3 | 1455.1 | 103.7 | 1.7 | 48.5 | 129.3 | 25.4 |
| 14.8 | f | 43.0 | 161 | 16.6 | III | pJIA RF− | 8.8 | MTX | high | 1460 | 34.0 | 1489.0 | 103.0 | 2.4 | 55.6 | 129.0 | 37.5 |
| 17.6 | m | 66.0 | 167 | 23.7 | IV | ERA | 57.4 | MTX | high | 2550 | 38.6 | 2131.9 | 214.0 | 3.2 | 41.9 | 129.0 | 42.0 |
| 16.8 | m | 58.0 | 178 | 18.3 | IV | undiff | 10.9 | AINS | mod. | 2190 | 37.8 | 2341.7 | 138.6 | 2.4 | 39.8 | 110.7 | 33.3 |
| 15.7 | f | 58.0 | 165 | 21.3 | IV | oJIA | 173.2 | MTX | mod. | 700 | 12.1 | 1516.0 | 151.1 | 2.6 | 33.5 | 100.4 | 22.1 |
| 10.1 | f | 24.0 | 133 | 13.6 | II | oJIA | 101.1 | MTX | mod. | 990 | 41.3 | 1062.6 | 134.5 | 5.6 | 59.5 | 122.4 | 23.3 |
| JIA: juvenile idiopathic arthritis; oJIA: oligoarticular JIA; pJIA RF−: rheumatoid factor-negative (RF−) polyarticular JIA; ERA: enthesitis-related arthritis; undiff : undifferentiated ; DMARDs: disease-modifying antirheumatic drugs; MTX: methotrexate; NSAIDs: nonsteroidal anti-inflammatory drugs; IPAQ: International Physical Activity Questionnaire ; mod. : moderate ; MFO : maximal fat oxydation rate ; bm : body mass ; HR : heart rate. | | | | | | | | | | | | | | | | | |
